# A short-term longitudinal study on the development of moral disengagement among schoolchildren: the role of collective moral disengagement, authoritative teaching, and student-teacher relationship quality

**DOI:** 10.3389/fpsyg.2024.1381015

**Published:** 2024-05-01

**Authors:** Marlene Bjärehed, Björn Sjögren, Robert Thornberg, Gianluca Gini, Tiziana Pozzoli

**Affiliations:** ^1^Department of Primary Teacher Education, Kristianstad University, Kristianstad, Sweden; ^2^Department of Behavioural Sciences and Learning, Linköping University, Linköping, Sweden; ^3^Department of Developmental Psychology and Socialisation, University of Padua, Padua, Italy

**Keywords:** moral disengagement, collective moral disengagement, authoritative teaching, student-teacher relationship quality, bullying, peer aggression

## Abstract

The aim of this study was to examine whether collective moral disengagement and authoritative teaching at the classroom level, and student-teacher relationship quality at the individual level, predicted individual moral disengagement among pre-adolescent students 1 year later. In this short-term longitudinal study, 1,373 students from 108 classrooms answered a web-based questionnaire on tablets during school, once in fifth grade (T1) and once in sixth grade (T2). The results showed, after controlling for T1 moral disengagement, gender, and immigrant background, that students with better student-teacher relationship quality at T1 were more inclined to score lower on moral disengagement at T2, whereas students in classrooms with higher levels of collective moral disengagement at T1 were more inclined to score higher on moral disengagement at T2. In addition, both collective moral disengagement and authoritative teaching were found to moderate the associations between student-teacher relationship quality at T1 and moral disengagement at T2. These findings underscore the importance of fostering positive relationships between students and teachers, as well as minimizing collective moral disengagement in classrooms. These measures may prevent the potential escalation of moral disengagement in a negative direction.

## Introduction

Although there exist different theories and research traditions of children’s moral development, most of them focus on how children progress in and increase their morality (Jensen, 2020; Killen and Smetana, 2022). However, as Bussey (2020) notes, there is a lack of research regarding children’s adherence to moral standards, including the moral standards–behavior gap. For instance, already by age three, children typically recognize moral transgressions, such as being mean to others, independently of authority figures and rules (for a meta-analysis, see Yoo and Smetana, 2022), yet schoolchildren still engage in immoral actions, such as bullying. Considering the pervasive moral socialization of children taking place at home and in school, and despite their own progress and advancement in moral development, including the understanding by the time they are of preschool age that bullying is wrong, why do schoolchildren engage in such immoral behavior?

Bandura’s social cognitive theory provides insights into this possible moral standard–behavior gap, highlighting the role of *moral disengagement* in justifying immoral behaviors (Bandura, 1999, 2002, 2016). Moral disengagement is a social cognitive process allowing individuals to justify harmful behaviors towards others, enabling them to act immorally without experiencing typical moral self-sanctions like remorse, guilt, or shame (Bandura, 2002, 2016). Previous research has established a clear link between moral disengagement and bullying (Killer et al., 2019; Thornberg, 2023), a pervasive issue affecting children and adolescents worldwide in school settings (Bradshaw et al., 2017; Cosma et al., 2020). Consequently, the development of moral disengagement emerges as a critical concern in the field of child development and education. Recognizing the pivotal role of moral development within educational contexts and the significant influence of school environments on students’ behavior (Eccles and Roeser, 2015), longitudinal research on predictors of moral disengagement within a school context is needed. However, existing longitudinal studies often treat moral disengagement as a predictor rather than investigating its antecedents (Thornberg, 2023). To address this gap, the aim of our study is to examine whether students’ moral disengagement in peer aggression is predicted by students’ perceptions of student-teacher relationships, the degree of authoritative teaching and collective moral disengagement within the classroom over the course of 1 year.

In Sweden, compulsory schooling comprises four stages: a pre-school class (age 6), lower elementary school (grades 1–3, ages 7–9), upper elementary school (grades 4–6, ages 10–12), and lower secondary school (grades 7–9, ages 13–15). In elementary school, students typically remain in a single classroom (homeroom) with the same classmates for most subjects, where one or two primary class teachers teach the majority of subjects, with only a few additional teachers (e.g., physical education, arts). Additionally, it is common for class teachers to stay with the same group of students from first to third grade (lower elementary teachers) and then from fourth to sixth grade (upper elementary teachers). Thus, the classroom, defined as the social setting in which students and teachers interact and influence each other’s attitudes and behaviors (Farmer et al., 2011; Hendricks et al., 2016), emerges as an important unit of analysis when examining factors influencing the development of moral disengagement in Swedish children.

In the current study, we focus on early adolescence and the last 2 years of the Swedish elementary school, from age 11 to age 12. Focusing on this age group is particularly important because bullying seems to be most prevalent during these years, both in Sweden (Friends, 2022) and elsewhere (Due et al., 2005). Furthermore, studying moral disengagement development at the beginning of adolescence is crucial because it’s a phase where individuals start to actively shape their sense of identity and values (Sawyer et al., 2012). This period offers a vital window for investigating the foundational aspects, precursors, and implications of morality, given its role in shaping lifelong ethical attitudes and behaviors (Malti et al., 2021). Further, our focus on the role of teachers and peers in the present study aligns with Bronfenbrenner’s (1979) conceptualization of the *microsystem*, and thus aims to explore how specific factors—on their own and in interaction—within a student’s immediate and direct environment influence the development of moral disengagement. Relationships with teachers and peers play a significant role in shaping a students’ development, influencing their beliefs, behaviors, psychological health, and social relationships (Hamre and Pianta, 2001; Cornelius-White, 2007; Farmer et al., 2019; Troop-Gordon et al., 2019).

### Moral disengagement theory

Within the framework of moral disengagement theory, Bandura (2016) described eight mechanisms through which moral disengagement occurs: through moral justification, using euphemistic language to soften destructive actions, comparing one’s actions favorably to others’, avoiding personal responsibility by shifting blame elsewhere, diffusing responsibility within a group, distorting or minimizing the consequences of one’s actions, dehumanizing others to justify mistreatment, and attributing blame to victims. By selectively using these moral disengagement mechanisms, individuals can avoid negative self-sanction, thereby increasing the likelihood of engaging in harmful behaviors.

A fundamental principle in the social cognitive theory is viewing humans as active agents who can intentionally influence their functioning and life circumstances (Bandura, 1986). Moral agency – the ability to refrain from immoral actions and act humanely - results from the interactions among personal factors (e.g., cognition, emotions), social and environmental factors (e.g., family, peer group reactions), and behaviors (e.g., aggression). In other words, behavior and cognition - such as moral disengagement - are partly the result of socialization processes within different socio-cultural contexts (Bandura, 1999). Indeed, previous research has shown that poor parental supervision and monitoring (Campaert et al., 2018), rejecting parenting, and neighborhood impoverishment (Hyde et al., 2010) positively predict moral disengagement over time. Regarding the peer context, Fontaine et al. (2014) revealed that peer rejection predicted subsequent moral disengagement, while Caravita et al. (2014) found that in early adolescence (but not in late childhood), Italian students became more like their friends in terms of level of moral disengagement over time. In a recent study, Korean elementary school students were also found to become more like their friends concerning moral disengagement over time (Kim et al., 2024).

### Collective moral disengagement

Social cognitive theory does not only include personal agency but also collective agency as a central part of the self-regulatory process (Bandura, 2002). Therefore, moral disengagement can also be considered a group characteristic (White et al., 2009; Bandura, 2016). Specifically, *collective moral disengagement* is “an emergent group-level property arising from the interactive, coordinative, and synergistic group dynamics” (White et al., 2009, p. 43). In schools, collective moral disengagement may emerge at the school or classroom level, whereas it may operate more broadly in other contexts (e.g., the community; Bussey, 2020). At the classroom level, it captures the shared beliefs of students about the extent to which moral disengagement mechanisms are common among classmates (Gini et al., 2014b). Previous research from Sweden (Thornberg et al., 2021; Bjärehed, 2022) and other European countries (Gini et al., 2014b; Kollerová et al., 2018) has demonstrated that students in classrooms characterized by higher levels of collective moral disengagement are more likely to engage in peer aggression and bullying. With the particular interest of the current study, a meta-analytical review (Luo and Bussey, 2023) identified collective moral disengagement as one of the environmental correlates of moral disengagement, suggesting a positive association between the two constructs. In other words, in classrooms with higher levels of collective moral disengagement, students are also more prone to enlist moral disengagement mechanisms (Gini et al., 2022).

Furthermore, collective moral disengagement has been found to moderate the association between individual moral disengagement and aggressive behaviors. For instance, in one Swedish study, Sjögren et al. (2021a) found that students more often reinforced or assisted in bullying situations if they belonged to classrooms with higher levels of collective moral disengagement and, at the same time, scored higher on individual moral disengagement. This aligns with the social cognitive theory, which suggests that collective processes interact with and influence individual behavior. Whether or not collective moral disengagement influences the development of individual students’ tendency to moral disengagement is still not well known. To the best of our knowledge, the present study is the first to examine whether or not collective moral disengagement at the classroom level predicts students’ moral disengagement over time.

### Authoritative teaching

The concept of authoritative parenting was introduced by Baumrind (1966), in her influential work on parenting styles, and has been negatively linked with moral disengagement in young adults (Di Pentima et al., 2023). Although this theory primarily addresses parents and child-rearing, scholars have highlighted the parallels between parents and teachers in their shared objective of fostering positive development and learning in their children and students (Wentzel, 2002; Walker, 2009; Ertesvåg, 2011). Teachers play a significant role in shaping the growth of young individuals and can serve as influential socialization agents in their students’ personal and social development (Farmer et al., 2011, 2019). *Authoritative teaching* refers to high levels of support (responsiveness) and structure (demandingness). While support includes warmth, care, responsiveness, and open communication, structure is about high expectations, demandingness, and strict but fair enforcement of school rules and classroom order (Walker, 2009; Gregory et al., 2010; Thornberg et al., 2018). Finne et al. (2018) have argued that an authoritative teaching style can counteract a destructive classroom power structure and foster moral engagement among students. Accordingly, authoritative schools and teaching have been linked to greater academic achievement in the United States (Dever and Karabenick, 2011) and less bullying and victimization among Swedish children (Thornberg et al., 2018; Kloo et al., 2023), Chinese adolescents (Wang et al., 2022), and American adolescents (Gregory et al., 2010; Cornell et al., 2015; Lau et al., 2018). However, less is known about the role of authoritative teaching for the development of students’ moral disengagement. Thus, the current study is the first to investigate whether authoritative teaching at the classroom level predicts students’ moral disengagement over time.

### Student-teacher relationship quality

A warm, caring, and supportive *student-teacher relationship* is a vital part of authoritative teaching. However, the quality of the relationship between an individual student and the teacher does not necessarily align with the overall teaching style at the classroom level. Previous research has shown that a higher quality relationship between a student and teacher (warmer, more caring and supportive, more respectful interaction patterns) is associated with less bullying and peer victimization (for a meta-analysis, see ten Bokkel et al., 2023). Although it is our understanding that no studies have examined the predictive role of student-teacher relationship quality on student’s moral disengagement within a longitudinal design, a few cross-sectional studies on bullying and peer victimization have included both moral disengagement and student-teacher relationship quality (or aspects of the latter). For instance, one Swedish study showed that students demonstrating higher moral disengagement in conjunction with poorer student-teacher relationship quality were more prone to reinforce in bullying situations (Sjögren et al., 2021b). Another study on ethnic bullying in Italy showed that closeness to teachers might restrain morally disengaged children from bullying (Iannello et al., 2021).

Furthermore, moral disengagement has been tested as a *mediator* between different school factors (e.g., school climate, teachers’ responses to bullying, student–student relationships) and bullying and aggression (e.g., Campaert et al., 2017; Ivaniushina and Alexandrov, 2022; Gao et al., 2023), suggesting that school factors may influence students’ moral disengagement. Concerning student-teacher relationships, one recent cross-sectional study examined whether moral disengagement mediated the association between student-teacher relationship quality and classroom incivility (Gao et al., 2024). Classroom incivility here refers to student behaviors that negatively impact the learning environment, encompassing everything from minor disturbances to physical violence. The findings from this study showed that students with poorer student-teacher relationship quality scored also higher in moral disengagement, which in turn was linked to more classroom incivility behaviors. The authors suggest that a warm, caring, and supportive teacher may model positive communication patterns and behaviors, possibly helping students to “respect and understand others, strengthen their moral and rule constraints, and thereby reduce their levels of moral disengagement” (Gao et al., 2024, p. 508). Nevertheless, the cross-sectional design did not allow conclusions about the directionalities of these associations. The present study is the first to examine whether student-teacher relationship quality at the individual level predicts students’ moral disengagement over time.

### The present study

The present study aimed to examine whether collective moral disengagement and authoritative teaching at the classroom level and student-teacher relationship quality at the individual level predicted individual moral disengagement among pre-adolescent students 1 year later. Given that previous studies suggest there are gender differences in moral disengagement (Caravita et al., 2012; Thornberg et al., 2023; Gao et al., 2024), and considering that a student’s tendency to morally disengage may vary due to differing socialization practices across cultures (Bussey, 2020), we included gender and immigrant background as control variables, along with the initial level of moral disengagement. Because Bandura (2016) argues that moral disengagement is “manifested differently depending on the sphere of activity” (p. 26), we delimited classroom collective moral disengagement and students’ moral disengagement in this study to the activity of peer aggression, including bullying and other forms of mean, unwanted or harmful behaviors toward peers. Thus, we did not study students’ proneness to morally disengage in general but how inclined they were to morally disengage when considering peer aggression.

Drawing on social cognitive theory, which posits that behaviors and cognitions are, in part, the result of socialization processes within different socio-cultural contexts (Bandura, 1999), and empirical evidence from cross-sectional studies that collective moral disengagement at the classroom level is negatively associated with defending behavior (Gini et al., 2015; Kollerová et al., 2018), and positively linked with peer aggression (Gini et al., 2015), bullying perpetration (Kollerová et al., 2018; Bjärehed et al., 2021; Thornberg et al., 2021), siding with peer aggressors (Sjögren et al., 2021a), and individual moral disengagement (Luo and Bussey, 2023), we hypothesized that greater collective moral disengagement at the classroom level would predict greater individual moral disengagement 1 year later (*Hypothesis 1*).

Based on previous cross-sectional findings showing that authoritative parenting is negatively linked with moral disengagement (Di Pentima et al., 2023), that higher school structure (a dimension of an authoritative school construct) is associated with less moral disengagement (Ivaniushina and Alexandrov, 2022), that authoritative teaching is negatively linked with bullying and pro-bullying behaviors (Lau et al., 2018; Thornberg et al., 2018; Kloo et al., 2023), and that individually perceived authoritative school climate is negatively linked with moral disengagement (Teng et al., 2020), we hypothesized that greater authoritative teaching at the classroom level would predict less moral disengagement 1 year later (*Hypothesis 2*).

With reference to research showing that greater student-teacher relationship quality decreases the risk of bullying perpetration (ten Bokkel et al., 2023) and cross-sectional studies showing a negative correlation between student-teacher relationship quality and moral disengagement (Sjögren et al., 2021b; Gao et al., 2024), we hypothesized that greater student-teacher relationship quality at the student level would predict less moral disengagement 1 year later (*Hypothesis 3*).

Given that social cognitive theory (Bandura, 1986) emphasizes the interplay between personal, behavioral, and environmental factors, and that previous research has not yet studied whether collective moral disengagement or authoritative teaching at the classroom level interacts with student-teacher relationship quality at the individual level to predict moral disengagement, we examined potential cross-level interaction effects in an exploratory manner.

## Methods

### Participants and procedure

Data were collected within a four-year longitudinal project that examined individual and classroom social and moral correlates of school bullying among Swedish school children from upper elementary school to the second year of lower secondary school. The overarching project started in the academic year 2015/2016. A total of 2,448 fifth-grade students in 64 schools were invited to participate in the current study. We used a strategic selection methodology which meant that the sample included students from different socio-geographical areas in Sweden (e.g., rural areas, small towns, and cities). Out of the original sample, 1,623 students filled in the questionnaire in grade 5 (51% girls). Reasons for non-participation included failure to submit parental consent (785 students) or absence on the day of data collection (40 students).

The students answered a web-based questionnaire on tablets in their regular classroom setting on two occasions: the first time in grade 5 and the second 1 year later in grade 6. Of those who answered the questionnaire in both grade 5 and grade 6, the final sample consisted of data from 1,373 students (grade 5, *M* = 11.5 and *SD* = 0.3), nested in 108 classrooms. For attrition analyses, we assessed whether students who continued their participation (*n* = 1,373) from fifth to sixth grade differed from those who only participated in fifth grade (*n* = 250) in terms of their levels of individual moral disengagement, student-teacher relationship quality, and perceptions of authoritative teaching and collective moral disengagement in their classroom in fifth grade. Independent *t* tests showed that there were no group differences in any of these variables in fifth grade. Written informed parental consent and student assent were obtained from all participants, and no incentives were provided for participation. During each session, either a member of the research team or a teacher was present to explain the study procedures and aid participants as needed. This assistance included providing reading support and clarifying specific items or words on the questionnaire. Participants were also informed of their right to withdraw from the study at any time, with assurance that their individual responses would remain inaccessible to both parents and school personnel. Additionally, participants were instructed to sit at a distance from one another to prevent viewing each other’s responses. On average, participants took approximately 30 min to complete the questionnaire.

### Measures

#### Moral disengagement

Moral disengagement at the individual level was measured with an 18-item self-report scale in grade 5 (T1) and grade 6 (T2). This scale was specifically developed in Swedish for the overarching longitudinal project to capture moral disengagement in peer aggression. Previous scales have commonly addressed either moral disengagement in more general antisocial behavior (Bandura et al., 1996), or specifically in bullying situations (Hymel et al., 2005; Thornberg and Jungert, 2014). The scale used in the current study has previously demonstrated adequate psychometric properties among Swedish school children (Thornberg et al., 2019; Bjärehed et al., 2021; Sjögren et al., 2021a; Bjärehed, 2022).

The students were asked to rate the extent to which they agreed (1 = *Strongly disagree* to 7 = *Strongly agree*) with each of the 18 items (e.g., “If you cannot be like everybody else, it is your fault if you get bullied or frozen out.” or “If my friends begin to tease a classmate, I cannot be blamed for being with them and teasing that person too.”) The scale captured all eight moral disengagement mechanisms described by Bandura (2016). CFAs with the MLM estimator displayed adequate fit: (grade 5: *χ*^2^ (135) = 392.018, *p* < 0.001, CFI = 0.90, RMSEA = 0.067; 90% CI [0.059, 0.074], SRMR = 0.055; grade 6: *χ*^2^ (135) = 405.179, *p* < 0.001, CFI = 0.91, RMSEA = 0.063; 90% CI [0.056, 0.070], SRMR = 0.046). For the current sample, Cronbach’s α was 0.87 in the fifth and 0.89 in the sixth grades. Therefore, the mean score of all items was computed as an index for moral disengagement. This measured the students’ overall tendency to morally disengage in peer aggression situations.

#### Student–teacher relationship quality

Student-teacher relationship quality (STRQ) was measured in grade 5 (T1) with a 13-item self-report scale, specifically developed in Swedish for the overarching project. The scale has demonstrated adequate psychometric properties among Swedish school children (Forsberg et al., 2023). The students were asked to rate the extent to which they agreed (1 = *Strongly disagree* to 7 = *Strongly agree*) with each of the 13 items. Seven items were positively worded to capture positive student–teacher relationship qualities, and six were negatively worded to capture negative student–teacher relationship qualities (as perceived by the student). Some example items are: “My teachers really care about me” (positive STRQ) and “My teachers do not like me” (negative STRQ). The negatively worded items were reversed and the mean score of all thirteen items was computed as an index variable. Thus, higher values on the index variable represent a positive relationship. CFA with the MLM estimator (two factors and accounting for the nested structure) displayed adequate fit: *χ*^2^ (64) = 364.825, *p* < 0.001, CFI = 0.95, RMSEA = 0.083; 90% CI [0.074, 0.091], SRMR = 0.048. Cronbach’s α for the whole scale (with reversed items) was 0.94.

#### Collective moral disengagement

Collective moral disengagement (CMD) was measured in grade 5 (T1) with an 18-item self-report scale, specifically developed in Swedish for the overarching project, that has demonstrated adequate psychometric properties among Swedish school children (Alsaadi et al., 2018; Bjärehed et al., 2021; Sjögren et al., 2021a; Bjärehed, 2022). The scale consisted of the same items as those measuring individual moral disengagement. However, to capture the collective dimension, this scale used the same procedure as in Gini et al.’s (2014b) classroom CMD scale and asked: “In your class, how many students think that…?” which the students then answered by selecting one of the following response categories: “None,” “About one quarter,” “About half,” “About three quarters,” and “Everyone.” At the individual level, the scale represented the individual’s perception of the degree to which moral disengagement was shared among peers in their classroom (referred to as *student-perceived collective moral disengagement*; Gini et al., 2014a). CFA with the MLM estimator (accounting for the nested structure) displayed adequate fit: *χ*^2^ (135) = 558.574, *p* < 0.001, CFI = 0.91, RMSEA = 0.070; 90% CI [0.064, 0.077], SRMR = 0.043. Cronbach’s α for the current sample was 0.92. Collective moral disengagement as a classroom-level construct was obtained by calculating the average score of all classroom members’ mean scores.

#### Authoritative teaching

To measure authoritative teaching in grade 5 (T1), we used two subscales from the 15-item Authoritative Classroom Climate Scale (Thornberg et al., 2018). This scale was specifically developed in Swedish for the overarching project and has demonstrated adequate psychometric properties among Swedish school children (Thornberg et al., 2018). The scale consists of two subscales of authoritative teaching: teacher support (4 items, e.g., “our teachers really care about the students,” “our teachers really give the students good help and support”) and teacher structure (4 items, e.g., “our teachers bring order and undisturbed working atmosphere in the classroom,” “our teachers make clear demands on students”). The students were asked to rate the extent to which they agreed (1 = *Strongly disagree* to 7 = *Strongly agree*) with the eight statements. CFA with the MLM estimator (two factors and accounting for the nested structure) displayed adequate fit: *χ*^2^ (19) = 68.751, *p* < 0.001, CFI = 0.97, RMSEA = 0.044; 90% CI [0.036, 0.052], SRMR = 0.021. Cronbach’s α for the current sample was 0.91. As with the scale measuring collective moral disengagement, we calculated each student’s mean and then averaged the scores for each classroom.

#### Control variables

Gender and immigrant background were included as control variables at the individual level. The students were asked whether they identified as a girl or a boy (girl = 0, boy = 1). For immigrant background, defined as not being born in Sweden or having two foreign-born parents, the students were asked whether they and their parents were born in Sweden. From these answers, a dummy variable was created indicating whether the students had a Swedish or immigrant background (0 = Swedish background, 1 = Immigrant background).

### Analyses

The students (*N* = 1,373) were nested within classrooms (*M* = 108). Thus, multilevel modeling techniques were used to analyze the data. This allowed us to disentangle individual-level and classroom-level effects on grade 6 moral disengagement (MD6). The individual-level variables were student-teacher relationship quality (STRQ5), immigrant background, gender, and moral disengagement (MD5), all reported in grade 5. The classroom-level variables examined were authoritative teaching (AUTH5) and collective moral disengagement (CMD5), also reported in grade 5.

First, we estimated an unconditional model with a random intercept. In this model, we estimated the overall classroom-level variance in moral disengagement (MD6). To test whether greater student-teacher relationship quality (STRQ5) predicted greater moral disengagement (MD6) 1 year later (*Hypothesis 3*), we added the individual-level variables as fixed effects (Model 1). Thus, this model examined the influence of student-teacher relationship quality in grade 5 on grade 6 moral disengagement while controlling for grade 5 moral disengagement, gender, and immigrant background.

To test whether greater authoritative teaching (*Hypothesis 2*) and less collective moral disengagement (*Hypothesis 1*) at the classroom level would predict less moral disengagement 1 year later, the two grade 5 classroom variables (AUTH5 and CMD5) were added as fixed effects. That is, in model 2, we examined the contribution of authoritative teaching and collective moral disengagement on grade 6 moral disengagement, over and above the individual’s student-teacher relationship quality (STRQ5), and the control variables. Lastly, in Model 3, we added the four cross-level interaction effects between moral disengagement, student-teacher relationship quality, authoritative teaching, and collective moral disengagement (MD5 × AUTH5, STRQ5 × AUTH5, IMD5 × CMD5, and STRQ5 × CMD5). This final model examined whether the effects of the individual-level variables differed depending on different classroom levels of authoritative teaching and collective moral disengagement.

We examined model fit improvement for each new model to assess the added variables’ explanatory value to the overall model. To help interpretation, the individual-level variables (except immigrant background and gender) were grand-mean centered, whereas the classroom-level variables were centered around the mean of all classrooms. All multilevel regression analyses were conducted in RStudio (version 2023.06.2) with the package *lme4* and the restricted maximum likelihood estimator (REML). All models were refitted with the maximum likelihood estimator (ML) to examine model improvement.

## Results

### Descriptive statistics and correlations

Descriptive statistics and correlations of the individual-level and classroom-level variables are presented in Tables 1, 2. Moral disengagement in grade 5 was positively correlated with grade 6 moral disengagement (*r* = 0.54, *p* < 0.001). In contrast, student-teacher relationship quality in grade 5 was negatively correlated with moral disengagement in grade 5 (*r* = −0.42, *p* < 0.001) and grade 6 (*r* = −0.38, *p* < 0.001). In other words, students with better student-teacher relationship quality in the fifth grade were more likely to score lower on moral disengagement in both grade 5 and grade 6. At the classroom level, collective moral disengagement in grade 5 was positively associated with the class mean of moral disengagement in grade 6 (*r* = 0.61, *p* < 0.001), whereas authoritative teaching in grade 5 was negatively associated with the class mean of moral disengagement in grade 6 (*r* = −0.50, *p* < 0.001). Thus, classrooms with greater authoritative teaching and classrooms with less collective moral disengagement were more likely to have a lower class mean of moral disengagement than other classrooms 1 year later. In addition, there was a negative correlation at the classroom level between authoritative teaching and collective moral disengagement in grade 5 (*r* = −0.71, *p* < 0.001), which means that collective moral disengagement tended to be lower in classrooms where teachers displayed greater authoritative teaching.

**Table 1:** Correlations, means, and standard deviations for individual-level variables.

	1	2	3	*M*	*SD*
1. Moral disengagement (T2)	–	–	–	1.41	0.60
2. Moral disengagement (T1)	0.54^*^	–	–	1.40	0.62
3. Student-teacher relationship quality	−0.38^*^	−0.42^*^	–	6.09	1.10

^*^p < 0.001. Number of students = 1373.

**Table 2:** Correlations, means, and standard deviations for classroom-level variables.

	1	2	3	Class *M*	*SD*
1. Moral disengagement (T2)^a^	–	–	–	1.41	0.24
2. Authoritative teaching (T1)	−0.50^*^	–	–	5.72	0.61
3. Collective moral disengagement (T1)	0.61^*^	−0.71^*^	–	1.51	0.24

^*^p < 0.001. Number of classrooms = 108. ^a^Classroom-mean of student moral disengagement.

### Multilevel regression analyses

Calculations of the intraclass coefficient (ICC) revealed variation at the classroom level, accounting for about 9% of the total variance in moral disengagement in the sixth grade. Therefore, the use of multilevel modeling was justified. As described earlier, Model 1 examined the influence of student-teacher relationship quality in grade 5 on grade 6 moral disengagement while controlling for grade 5 moral disengagement, gender, and immigrant background (*Hypothesis 3*). In Model 2, *Hypotheses 1* and 2 were examined. In the third model, interaction effects were added and examined in an exploratory manner. Reported coefficients, as presented in Table 3 and in the text, are unstandardized and thus indicate the expected change in moral disengagement for each unit of change in the independent variable. The results of the multilevel modeling are summarized in Table 3.

**Table 3:** Multilevel estimates for models predicting student moral disengagement in grade 6.

	Unconditional		Model 1		Model 2		Model 3	
	Est.	*SE*	Est.	*SE*	Est.	*SE*	Est.	*SE*
*Student level*								
Gender			0.106^***^	0.03	0.105^***^	0.03	0.109^***^	0.03
Background			0.012	0.04	−0.002	0.04	0.020	0.04
Moral disengagement (MD5)			0.426^***^	0.02	0.418^***^	0.02	0.373^***^	0.03
Student teacher-relationship quality (STRQ5)			−0.101^***^	0.01	−0.096^***^	0.01	−0.096^***^	0.02
*Classroom level*								
Authoritative teaching (AUTH5)					0.045	0.04	0.054	0.04
Collective moral disengagement (CMD5)					0.323^**^	0.10	0.285^**^	0.09
*Cross-level interactions*								
MD5*AUTH5							−0.263^***^	0.05
STRQ5*AUTH5							−0.125^***^	0.03
MD5*CMD5							−0.192	0.14
STRQ5*CMD5							−0.340^***^	0.08
*Random effects*								
Intercept		0.033		0.012		0.009		0.006
Residual		0.331		0.234		0.235		0.226
AIC		2468.3		1964.7		1956.7		1900.2
Likelihood ratio test			Chisq(4) = 511.63^***^		Chisq(2) = 11.96^**^		Chisq(4) = 64.575^***^	
ICC	0.091							
Number of students	1373							
Number of classrooms	108							

^**^p < 0.01, ^***^p < 0.001. Gender: Girl = 0; Background: Swedish ethnic background = 0; Est., unstandardized coefficient; SE, standard error; ICC, intraclass coefficient; AIC, Akaike information criterion.

At the individual level, our results revealed that girls (*b* = 0.109, SE = 0.03, *p* < 0.001) and students who reported less moral disengagement (*b* = 0.373, SE = 0.03, *p* < 0.001) and greater student-teacher relationship quality in grade 5 (*b* = −0.096, SE = 0.02, *p* < 0.001) were inclined to score lower on moral disengagement in grade 6 (see Table 3, Model 3). In other words, we found support for our third hypothesis, as the quality of student-teacher relationship at the individual level negatively predicted students’ moral disengagement 1 year later, even when controlling for their moral disengagement in grade 5 and other variables in the model.

**Figure 1:**
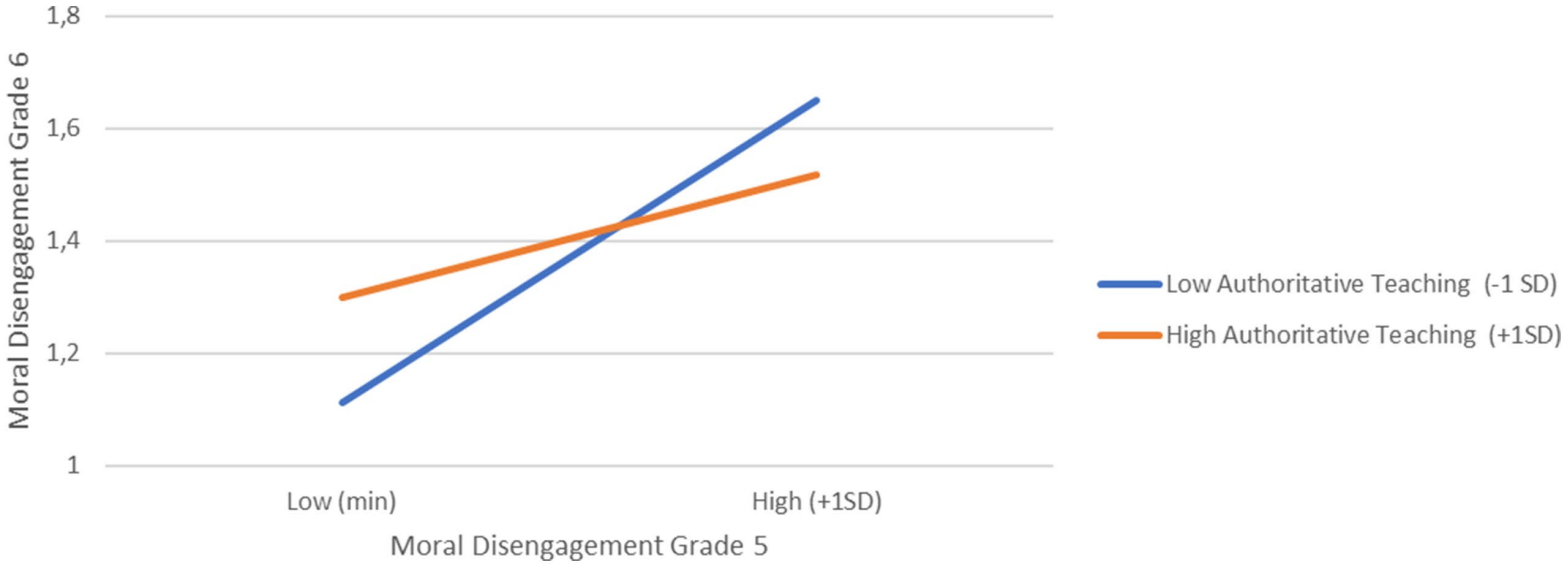
Cross-level interaction between moral disengagement and authoritative teaching predicting moral disengagement in grade 6.

**Figure 2:**
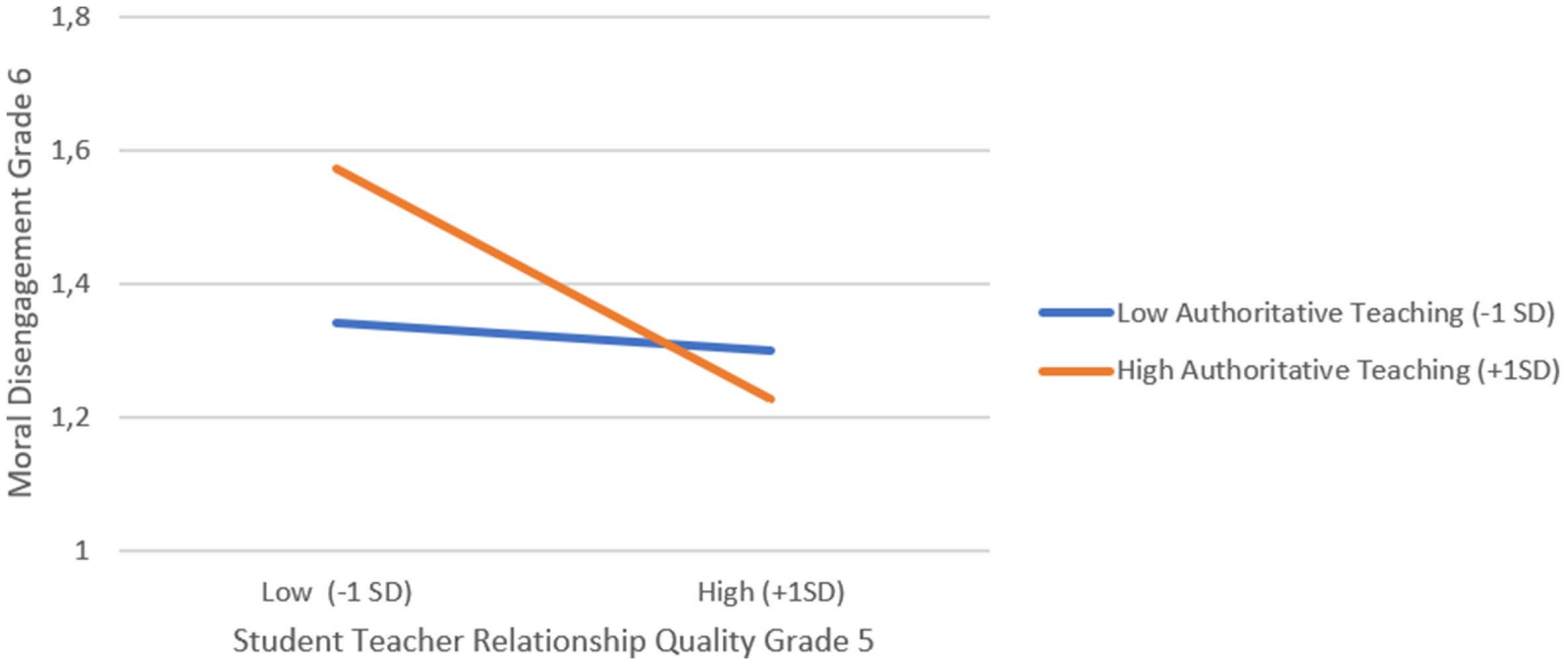
Cross-level interaction between student-teacher relationship quality and authoritative teaching predicting moral disengagement in grade 6.

**Figure 3:**
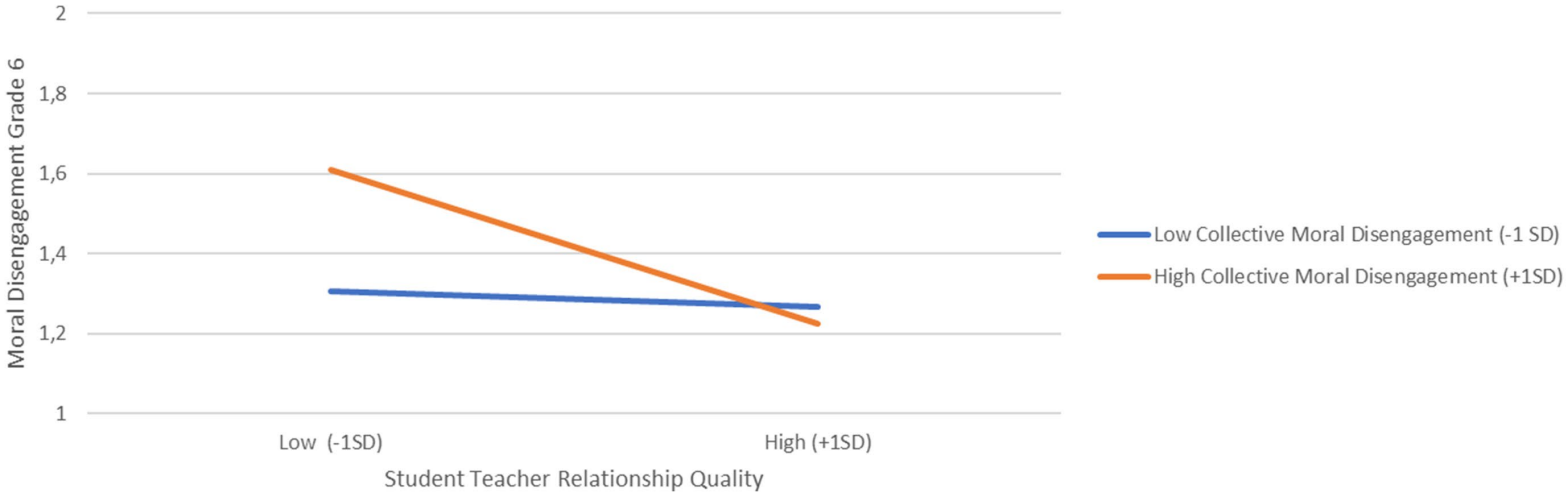
Cross-level interaction between student-teacher relationship quality and collective moral disengagement predicting moral disengagement in grade 6.

Additionally, we found support for our first hypothesis, as classroom levels of collective moral disengagement in grade 5 positively predicted individual students’ moral disengagement in grade 6 (*b* = 0.285, SE = 0.09, *p* < 0.01). Thus, students in classrooms characterized by higher levels of collective moral disengagement were more likely to score higher on moral disengagement 1 year later compared to students in classrooms with lower levels of collective moral disengagement in grade 5. Contrary to *Hypothesis 2*, authoritative teaching in grade 5 did not significantly predict moral disengagement in grade 6. However, interaction effects were found for three of the four interactions tested (see Figures 1–3).

First, there was a significant interaction between students’ moral disengagement and authoritative teaching in grade 5 (*b* = −0.26, SE = 0.05, *p* < 0.001). To interpret these significant interaction effects, we computed simple slopes (see Preacher et al., 2006) for low (–1SD) and high (+1SD) levels of authoritative teaching. As shown in Figure 1, the effect of moral disengagement in grade 5 on moral disengagement in grade 6 was stronger in classrooms characterized by low authoritative teaching (*b*_low_ = 0.53, *p* < 0.001) compared with classrooms characterized as high in authoritative teaching (*b*_high_ = 0.21, *p* < 0.001). Second, and as shown in Figure 2, we found a significant interaction between student-teacher relationship quality and authoritative teaching in grade 5 (*b* = −0.13, SE = 0.03, *p* < 0.001). Simple slope analysis for low (−1SD) and high classroom levels (+1SD) of authoritative teaching showed that in classrooms with high authoritative teaching, better student-teacher relationship quality was associated with less moral disengagement in the sixth grade (*b*_high_ = −0.17, *p* < 0.001), whereas in classrooms with low levels of authoritative teaching, grade 6 moral disengagement did not vary as a function of student-teacher relationship quality (*b*_low_ = −0.02, *p = 0*.31). Lastly, and as illustrated in Figure 3, there was a significant interaction between student-teacher relationship quality and collective moral disengagement in grade 5 (*b =* −0.340, SE = 0.09, *p* < 0.001). The simple slope for low (–1SD) collective moral disengagement was not significant (*b_low_* = −0.02, *p = 0*.42), whereas the simple slope for high (+1SD) collective moral disengagement was negative and significant (*b*_high_ = −0.17, *p* < 0.001). That is, in classrooms with high levels of collective moral disengagement, better student-teacher relationship quality was associated with lower levels of moral disengagement 1 year later.

## Discussion

With reference to social cognitive theory (Bandura, 2016), children can develop their moral standards, self-regulatory processes, and behaviors but can also learn to use moral disengagement mechanisms from socialization agents such as teachers and peers in school. Understanding the antecedents of moral disengagement is crucial, given its well-established link to school bullying (Killer et al., 2019; Luo and Bussey, 2023; Thornberg, 2023) and peer aggression more generally (Gini et al., 2014a; Luo and Bussey, 2023). In alignment with social cognitive theory and reciprocal determinism (Bandura, 2016), this study offers insights into the complex interplay between personal factors, including cognitions, and social factors, that are transmitted through the influence of peers and teachers. It examines the impact of specific school microsystem factors (Bronfenbrenner, 1979) on the development of moral disengagement in pre-adolescence, specifically focusing on collective moral disengagement, authoritative teaching, and the quality of student-teacher relationships. To our knowledge, the present study has been the first to examine how these factors, uniquely and interactively, predict moral disengagement within a longitudinal design.

### Collective moral disengagement

As hypothesized, our study revealed that students in classrooms with higher levels of collective moral disengagement in fifth grade were more prone to endorse morally disengaged beliefs about peer aggression 1 year later (*Hypothesis 1*). Thus, collective moral disengagement is a contextual factor that affects not only behaviors such as bullying (Gini et al., 2014b; Kollerová et al., 2018; Thornberg et al., 2021; Bjärehed, 2022) but also socio-cognitive processes, such as an individual’s moral disengagement. This underscores the significant influence of the peer group on the development of moral disengagement among pre-adolescents. Our finding adds to previous research by suggesting that not only do friends tend to become more similar in moral disengagement levels over time in early adolescence (Caravita et al., 2014; Kim et al., 2024), but pre-adolescents who belong to a classroom with higher levels of collective moral disengagement are at an increased risk of developing greater individual moral disengagement.

While students’ moral disengagement is in itself a risk factor for peer aggression and bullying (Gini et al., 2014a; Luo and Bussey, 2023), and a predictor of later bullying perpetration (for a review see Thornberg, 2023), collective moral disengagement at the classroom level has also been shown to be associated with bullying behaviors, over and above individual moral disengagement (Bjärehed et al., 2021; Sjögren et al., 2021a). In addition to this existing research, and a recent longitudinal study showing that classroom collective moral disengagement explains between-classroom variability in later aggression (Gini et al., 2022), the current findings contribute to the literature by demonstrating that classroom collective moral disengagement also predicts students’ moral disengagement over time. In other words, collective moral disengagement at the classroom level can be linked to a negative development of students’ moral cognition and behavior.

### Authoritative teaching

Although authoritative teaching was negatively associated with classroom levels of moral disengagement 1 year later in the bivariate analysis, there was no significant direct effect of authoritative teaching in fifth grade on moral disengagement in the sixth grade in the final model (*Hypothesis 2*). One study has found that students perceiving school rules as transparent and fair are less likely to activate mechanisms of moral disengagement, resulting in decreased engagement in negative behaviors (Ivaniushina and Alexandrov, 2022). The inconsistency with our findings may be explained by the focus of our study on students’ collective perceptions of their teacher’s teaching style rather than individual students’ perceptions. Ivaniushina and Alexandrov’s study also included a slightly older sample (ages 12–15). Further, they focused on structure as a school characteristic, while our measure included both structure and support and specifically examined the teacher and classroom setting. It is also plausible that support and structure are differentially associated with moral disengagement. In a Swedish study with grade 4 students, Kloo et al. (2023) distinguished between these two dimensions of authoritative teaching; their results suggest that teacher support drives the negative association between authoritative teaching and bullying perpetration. For example, high teacher structure might impact bullying through reduced moral disengagement, as proposed by Ivaniushina and Alexandrov (2022), while a classroom characterized by support (i.e., warmth, open communication, and caring) might be directly linked to lower levels of bullying and victimization (Lau et al., 2018; Thornberg et al., 2018; Kloo et al., 2023). In addition to the impact of collective moral disengagement at the classroom level found in the present study, another reasonable explanation could be that students’ perception of their relationship quality with the teacher (Gao et al., 2024) is a more influential factor for their development and changes in moral disengagement over time than the overall teaching style at the classroom level. This suggestion is supported by the finding in our study that more positive, warm, and supportive student-teacher relationships predicted lower levels of moral disengagement.

### Student-teacher relationship quality

The current results suggest that a higher quality of student-teacher relationships may protect against subsequent increases in moral disengagement (*Hypothesis 3*). This indicates that how teachers establish and maintain relationships with their students, and how this relationship quality varies across their student cohort, may play an essential role in their students’ development of moral disengagement. This finding holds significance, particularly in light of the well-established connection between moral disengagement and later involvement in school bullying, as identified in other studies (Thornberg, 2023). In our study, students with more negative, less supportive, and less caring student-teacher relationships were more likely to exhibit elevated levels of moral disengagement in the sixth grade, regardless of their initial level of moral disengagement. This result expands upon previous cross-sectional findings demonstrating a positive link between student-teacher relationships and moral disengagement (Gao et al., 2024) that also incorporate two time points within a longitudinal design.

In Gao et al.’s study, the association between student-teacher relationships and moral disengagement was stronger in early adolescence (11–14 years) compared to middle adolescence (15–17 years). From a developmental standpoint, adolescents progressively gain independence, thus suggesting that the influence of adults, including teachers, on their socialization might diminish later in adolescence. Consistent with this notion, prior longitudinal studies have indicated that moral disengagement is influenced by factors in home environments until late childhood (Hyde et al., 2010), after which it becomes more influenced by peers (Caravita et al., 2014). Our study adds to this literature by suggesting that teachers are influential socialization agents impacting students’ tendency to morally disengage in pre-adolescence. Not only can higher student-teacher relationship quality be associated with less bullying (ten Bokkel et al., 2023) and peer aggression (Krause and Smith, 2023). According to our study it can also be associated with less moral disengagement. The literature, in turn, has linked low moral disengagement with less bullying (Killer et al., 2019; Thornberg, 2023) and peer aggression (Gini et al., 2014a; Luo and Bussey, 2023). Altogether, this could be interpreted in terms of what Bandura (2016) calls triadic codetermination, which means that “human functioning is a product of the interplay of personal influences, the behavior individuals engage in, and the environmental forces that impinge on them” (p. 6). Personal influences would here be moral disengagement, behavior would refer to bullying or peer aggression, and environmental forces the student-teacher relationship quality. Our study contributes to the literature by showing that student-teacher relationship quality negatively predicted moral disengagement 1 year later. Since our study focused on changes between the ages of 11 and 12, it would be desirable to include a more extended period in future studies, both with younger school children and those in later adolescence. This could provide insights into the potentially changing influence of student-teacher relationship quality on moral disengagement across different developmental stages.

### Cross-level interaction effects

In our study, three out of four tested cross-level interactions significantly predicted moral disengagement 1 year later: the interaction between moral disengagement and authoritative teaching, the interaction between student-teacher relationship quality and authoritative teaching, and the interaction between student-teacher relationship quality and collective moral disengagement.

Although we did not find that authoritative teaching at the classroom level predicted subsequent moral disengagement (when controlling for collective moral disengagement at the classroom level and student-teacher relationship quality, gender, immigrant background, and previous moral disengagement at the individual level), authoritative teaching was found to moderate the effects of moral disengagement and student-teacher relationship quality in grade 5 on subsequent moral disengagement. In classrooms with less authoritative teaching, the impact of moral disengagement on subsequent moral disengagement was more pronounced. This finding suggests that an authoritative teaching style can act as a buffer against a negative spiral of escalating moral disengagement over time, especially for students who already exhibit high levels of moral disengagement in grade 5.

Our findings further indicate that lower student-teacher relationship quality in grade 5 is associated with higher moral disengagement in grade 6. However, this association was only significant in classrooms with high levels of authoritative teaching. In other words, this result suggests that having poor student-teacher relationships when belonging to classrooms where teachers are high in authoritative teaching is a risk factor of moral disengagement. With reference to the self-categorization theory (Turner and Oakes, 1989; Abrams and Hogg, 1990), a possible explanation for our findings might be that the few students who had poor relationships with their teachers compared themselves with the majority of their classmates who had more positive relationships with their teachers due to the authoritative teaching style (teachers showed greater warmth, care, support, and responsiveness to students in general). It is plausible to assume that these everyday social comparisons in school increase the risk of developing a sense of non-belonging in the classroom context together with a more deviant social identity. As a part of their self-categorization process (Turner and Oakes, 1989), students with poor teacher relationships would be prone to develop a self-serving bias of favoring the social category they identify themselves with (e.g., anti-school, antisocial, or rejected in-group) while devaluating the majority group of others in the classroom and other peers whom they perceive belong to the same well-adjusted outgroup. These “upward comparisons” (Laninga-Wijnen et al., 2023) might, in this case, contribute to an antisocial trajectory and a greater need for moral disengagement to maintain positive self-esteem and avoid self-sanctions.

In the final regression model, collective moral disengagement at the classroom level and the quality of the student-teacher relationship at the individual level were uniquely linked to moral disengagement in sixth grade. Nevertheless, the extent to which student-teacher relationship quality predicted moral disengagement also depended on the levels of classroom collective moral disengagement. Previous studies have demonstrated that collective moral disengagement can act as a protective group property within the classroom, reducing the risk of bullying perpetration (Gini et al., 2014b; Kollerová et al., 2018; Thornberg et al., 2019; Thornberg et al., 2021; Bjärehed, 2022). Our study contributes to this body of literature by suggesting that students with high student-teacher relationship quality score low on subsequent moral disengagement independently of classroom levels of collective moral disengagement. In contrast, students with low student-teacher relationship quality tend to score higher on subsequent moral disengagement in classrooms with high levels of collective moral disengagement compared to students in classrooms with low collective moral disengagement. Thus, having a positive student-teacher relationship appears to be an important protective factor against moral disengagement. The interaction effect in our results suggest that students who had positive, warm, and supportive relationships with their teachers were also better equipped to resist the bad influence of classroom collective moral disengagement on their moral development. One year later they still showed low levels of moral disengagement despite belonging to a classroom with high collective moral disengagement.

### Limitations

The current study fills an important gap in the literature by examining the role played by specific school microsystem factors (Bronfenbrenner, 1979) on the development of moral disengagement in pre-adolescence. Nevertheless, some methodological limitations need to be addressed. First, all the studied variables were assessed using self-report measures. Self-reports are vulnerable to social desirability biases, and there may be a risk that students underreport their moral disengagement while exaggerating how common it is among classmates. Nevertheless, as both moral disengagement and collective moral disengagement relate to students’ perceptions and beliefs, self-reports may be the best way to capture these constructs. Further, some controversy exists about whether students should rate their teachers’ behaviors (den Brok et al., 2006), like authoritative teaching. Thus, future studies could examine whether the current findings hold when teacher reports and/or direct observations are used to capture authoritative teaching.

Although our study implemented a longitudinal design, only two time points over a relatively short period of 1 year were included. A more extensive longitudinal approach with additional time points could be employed to enhance the robustness of our findings. This would provide a more comprehensive understanding of the observed associations and enable us to capture potential developmental trends or variations over an extended period. In addition, changes in moral disengagement may also be influenced by several factors not included in the current study.

Furthermore, no classroom mean score of authoritative teaching was below 3.9 (max 7); for collective moral disengagement, no classroom score exceeded 2.6 (max 5). This suggests that our study compared classrooms characterized as relatively authoritative and with relatively low levels of collective moral disengagement. Consequently, caution is advised when generalizing findings to classrooms characterized by a lower degree of authoritative teaching and higher levels of collective moral disengagement. Finally, our study exclusively involved students in Swedish schools. Future studies should investigate the transferability of these findings to other countries and cultural contexts.

### Practical implications

The current findings have implications for teacher educators and school personnel. First, fostering a positive and supportive relationship between students and teachers seems crucial in minimizing a negative developmental spiral of moral disengagement in pre-adolescence. Teacher educators should, therefore, focus on equipping pre-service teachers with the skills and knowledge to build positive and supportive relationships with their students (Bouchard and Smith, 2017). Teachers who engage in authoritative teaching as a part of their bullying prevention strategy (see Lau et al., 2018; Thornberg et al., 2018; Kloo et al., 2023) need to make the effort to build warm, caring, and responsive relationships with all of the students in their classroom and include targeted actions for students they fail to reach and with whom they fail to develop supportive relationships. To prevent an adverse moral climate, pre-service teachers and teachers can also benefit from professional training on influencing group dynamics (Hymel et al., 2015). This training should include strategies that proactively prevent collective moral disengagement from emerging in the first place and promote a moral climate defined by active engagement and social responsibility. In line with the democratic mission (Swedish Education Act, SFS 2010:800, 2010), teachers could integrate discussions about ethics and moral decision-making into their ordinary lessons by making students aware of moral disengagement mechanisms and how these can contribute to explaining a range of negative, inhumane and aggressive behaviors, including bullying and peer aggression, delinquency/criminality, political oppression, terrorism, genocide, and war (Bandura, 2016). When students are encouraged to reflect on ethical considerations and to identify moral disengagement mechanisms, they might be more likely to develop a moral compass and resist moral disengagement.

## Data availability statement

The datasets generated for this study are available on request to the corresponding author.

## Ethics statement

The studies involving humans were approved by Regional Ethical Review Board at Linköping. The studies were conducted in accordance with the local legislation and institutional requirements. Written informed consent for participation in this study was provided by the participants’ legal guardians/next of kin.

## Author contributions

MB: Writing – original draft, Writing – review & editing. BS: Writing – original draft, Writing – review & editing. RT: Writing – original draft, Writing – review & editing. GG: Writing – review & editing. TP: Writing – review & editing.

## References

[ref1] Abrams D. Hogg M. A. (1990). Social identification, self-categorization, and social influence. Eur. Rev. Soc. Psychol. 1, 195–228. doi: 10.1080/14792779108401862

[ref2] Alsaadi S. Wänström L. Thornberg R. Sjögren B. Bjärehed M. Forsberg C. (2018) Paper presented at the 2018 annual meeting of the American Educational Research Association. Retrieved from the AERA Online Paper Repository. (Accessed April 16, 2024).

[ref3] Bandura A. (1986). Social Foundations of Thought and Action: A Social Cognitive Theory. New Jersey: Prentice-Hall.

[ref9001] Bandura A. Barbaranelli C. Caprara G. V. Pastorelli C. (1996). Mechanisms of moral disengagement in the exercise of moral agency. J. Pers. Soc. Psychol. 71, 364–374. doi: 10.1037/0022-3514.71.2.364

[ref4] Bandura A. (1999). Moral disengagement in the perpetration of inhumanities. Personal. Soc. Psychol. Rev. 3, 193–209. doi: 10.1207/s15327957pspr0303_3, PMID: 15661671

[ref5] Bandura A. (2002). Selective moral disengagement in the exercise of moral agency. J. Moral Educ. 31, 101–119. doi: 10.1080/0305724022014322

[ref6] Bandura A. (2016). Moral Disengagement: How People Do Harm and Live with Themselves. New York: Worth.

[ref7] Baumrind D. (1966). Effects of authoritative parental control on child behavior. Child Dev. 37, 887–907. doi: 10.2307/1126611

[ref9] Bjärehed M. (2022). Individual and classroom collective moral disengagement in offline and online bullying: a short-term multilevel growth model study. Psychol. Sch. 59, 356–375. doi: 10.1002/pits.22612

[ref10] Bjärehed M. Thornberg R. Wänström L. Gini G. (2021). Individual moral disengagement and bullying among Swedish fifth graders: the role of collective moral disengagement and pro-bullying behavior within classrooms. J. Interpers. Violence 36, NP9576–NP9600. doi: 10.1177/0886260519860889, PMID: 31282237

[ref11] Bouchard K. L. Smith J. D. (2017). Teacher–student relationship quality and children’s bullying experiences with peers: reflecting on the mesosystem. Educ. Forum 81, 108–125. doi: 10.1080/00131725.2016.1243182

[ref12] Bradshaw J. Crous G. Rees G. Turner N. (2017). Comparing children's experiences of schools-based bullying across countries. Child Youth Serv. Rev. 80, 171–180. doi: 10.1016/j.childyouth.2017.06.060

[ref13] Bronfenbrenner U. (1979). The Ecology of Human Development: Experiments by Nature and Design. Cambridge, MA: Harvard University Press.

[ref14] Bussey K. (2020). “Development of moral disengagement” in The Oxford Handbook of Moral Development. ed. Jensen L. A. (Oxford: Oxford University Press), 305–326.

[ref15] Campaert K. Nocentini A. Menesini E. (2017). The efficacy of teachers’ responses to incidents of bullying and victimization: the mediational role of moral disengagement for bullying. Aggress. Behav. 43, 483–492. doi: 10.1002/ab.21706, PMID: 28317120

[ref16] Campaert K. Nocentini A. Menesini E. (2018). The role of poor parenting and parental approval for children’s moral disengagement. J. Child Fam. Stud. 27, 2656–2667. doi: 10.1007/s10826-018-1097-1

[ref17] Caravita S. C. Gini G. Pozzoli T. (2012). Main and moderated effects of moral cognition and status on bullying and defending. Aggress. Behav. 38, 456–468. doi: 10.1002/ab.21447, PMID: 22898969

[ref18] Caravita S. C. Sijtsema J. J. Rambaran J. A. Gini G. (2014). Peer influences on moral disengagement in late childhood and early adolescence. J. Youth Adolesc. 43, 193–207. doi: 10.1007/s10964-013-9953-1, PMID: 23660831

[ref19] Cornelius-White J. (2007). Learner-centered teacher-student relationships are effective: a meta-analysis. Rev. Educ. Res. 77, 113–143. doi: 10.3102/003465430298563

[ref20] Cornell D. Shukla K. Konold T. (2015). Peer victimization and authoritative school climate: a multilevel approach. J. Educ. Psychol. 107, 1186–1201. doi: 10.1037/edu0000038

[ref21] Cosma A. Walsh S. D. Chester K. L. Callaghan M. Molcho M. Craig W.. (2020). Bullying victimization: time trends and the overlap between traditional and cyberbullying across countries in Europe and North America. Int. J. Public Health 65, 75–85. doi: 10.1007/s00038-019-01320-2, PMID: 31844919

[ref22] den Brok P. D. Bergen T. Brekelmans M. (2006). “Convergence and divergence between students’ and teachers’ perceptions of instructional behaviour in Dutch secondary education” in Contemporary Approaches to Research on Learning Environments: Worldviews. eds. Fisher D. F. Khine M. S. (Singapore: World Scientific Publishing), 125–160.

[ref23] Dever B. V. Karabenick S. A. (2011). Is authoritative teaching beneficial for all students? A multi-level model of the effects of teaching style on interest and achievement. Sch. Psychol. Q. 26, 131–144. doi: 10.1037/a0022985

[ref24] Di Pentima L. Toni A. Roazzi A. (2023). Parenting styles and moral disengagement in young adults: the mediating role of attachment experiences. J. Genet. Psychol. 184, 322–338. doi: 10.1080/00221325.2023.2205451, PMID: 37178171

[ref25] Due P. Holstein B. E. Lynch J. Diderichsen F. Gabhain S. N. Scheidt P.. (2005). Bullying and symptoms among school-aged children: international comparative cross sectional study in 28 countries. Eur. J. Pub. Health 15, 128–132. doi: 10.1093/eurpub/cki105, PMID: 15755782

[ref26] Eccles J. S. Roeser R. W. (2015). “School and community influences on human development” in Developmental Science: An Advanced Textbook. eds. Bornstein M. H. Lamb M. E.. 7th ed (New York: Psychology Press), 645–728.

[ref27] Ertesvåg S. K. (2011). Measuring authoritative teaching. Teach. Teach. Educ. 27, 51–61. doi: 10.1016/j.tate.2010.07.002

[ref28] Farmer T. W. Hamm J. V. Dawes M. Barko-Alva K. Cross J. R. (2019). Promoting inclusive communities in diverse classrooms: teacher attunement and social dynamics management. Educ. Psychol. 54, 286–305. doi: 10.1080/00461520.2019.1635020

[ref29] Farmer T. W. McAuliffe Lines M. Hamm J. V. (2011). Revealing the invisible hand: the role of teachers in children’s peer experiences. J. Appl. Dev. Psychol. 32, 247–256. doi: 10.1016/j.appdev.2011.04.006

[ref30] Finne J. Roland E. Svartdal F. (2018). Relational rehabilitation: reducing harmful effects of bullying. Nordic Stud. Educ. 38, 352–367. doi: 10.18261/issn.1891-2018-04-05

[ref31] Fontaine R. G. Fida R. Paciello M. Tisak M. S. Caprara G. V. (2014). The mediating role of moral disengagement in the developmental course from peer rejection in adolescence to crime in early adulthood. Psychol. Crime Law 20, 1–19. doi: 10.1080/1068316X.2012.719622

[ref32] Forsberg C. Sjögren B. Thornberg R. Hong J. S. Longobardi C. (2023). Longitudinal reciprocal associations between, student-teacher relationship quality and verbal and relational bullying victimization. Soc. Psychol. Educ. 27, 151–173. doi: 10.1007/s11218-023-09821-y

[ref33] Friends. (2022). Friendsrapporten 2022. 10 år av Barns Röster! [The Friend’s Report 2022. 10 Years of Children’s Voices!]. Available at: https://friends.se/uploads/2022/08/Friendsrapporten-2022_WEBB.pdf (Accessed April 16, 2024).

[ref34] Gao L. Kong F. Cui L. Feng N. Wang X. (2024). Teacher–student relationships and adolescents’ classroom incivility: a moderated mediation model of moral disengagement and negative coping style. Psychol. Sch. 61, 496–513. doi: 10.1002/pits.23064

[ref35] Gao L. Li X. Wu X. Wang X. (2023). Longitudinal associations among student-student relationship, moral disengagement, and adolescents' bullying perpetration. Sch. Psychol. 38, 337–347. doi: 10.1037/spq0000534, PMID: 36877462

[ref36] Gini G. Pozzoli T. Bussey K. (2014b). Collective moral disengagement: initial validation of a scale for adolescents. Eur. J. Dev. Psychol. 11, 386–395. doi: 10.1080/17405629.2013.851024

[ref37] Gini G. Pozzoli T. Bussey K. (2015). The role of individual and collective moral disengagement in peer aggression and by standing: a multilevel analysis. J. Abnorm. Child Psychol. 43, 441–452. doi: 10.1007/s10802-014-992, PMID: 25123080

[ref38] Gini G. Pozzoli T. Hymel S. (2014a). Moral disengagement among children and youth: a meta-analytic review of links to aggressive behavior. Aggress. Behav. 40, 56–68. doi: 10.1002/ab.21502 24037754

[ref39] Gini G. Thornberg R. Bussey K. Angelini F. Pozzoli T. (2022). Longitudinal links of individual and collective morality with adolescents’ peer aggression. J. Youth Adolesc. 51, 524–539. doi: 10.1007/s10964-021-01518-9, PMID: 34661788 PMC8881436

[ref40] Gregory A. Cornell D. Fan X. Sheras P. Shih T.-H. Huang F. (2010). Authoritative school discipline: high school practices associated with lower bullying and victimization. J. Educ. Psychol. 102, 483–496. doi: 10.1037/a0018562

[ref41] Hamre B. K. Pianta R. C. (2001). Early teacher–child relationships and the trajectory of children's school outcomes through eighth grade. Child Dev. 72, 625–638. doi: 10.1111/1467-8624.00301, PMID: 11333089

[ref42] Hendricks M. M. H. G. Mainhard M. T. Boor-Klip H. J. Cillessen A. H. M. Brekelmans M. (2016). Social dynamics in the classroom: teacher support and conflict and the peer ecology. Teach. Teach. Educ. 53, 30–40. doi: 10.1016/j.tate.2015.10.004

[ref43] Hyde L. W. Shaw D. S. Moilanen K. L. (2010). Developmental precursors of moral disengagement and the role of moral disengagement in the development of antisocial behavior. J. Abnorm. Child Psychol. 38, 197–209. doi: 10.1007/s10802-009-9358-5 19777337 PMC2858331

[ref44] Hymel S. McClure R. Miller M. Shumka E. Trach J. (2015). Addressing school bullying: insights from theories of group processes. J. Appl. Dev. Psychol. 37, 16–24. doi: 10.1016/j.appdev.2014.11.008

[ref45] Hymel S. Rocke-Hendersson N. Bonanno R. A. (2005). Moral disengagement: a framework for understanding bullying among adolescents. J. Soc. Sci. 8, 1–11.

[ref46] Iannello N. M. Camodeca M. Gelati C. Papotti N. (2021). Prejudice and ethnic bullying among children: the role of moral disengagement and student-teacher relationship. Front. Psychol. 12:713081. doi: 10.3389/fpsyg.2021.713081, PMID: 34539514 PMC8446265

[ref47] Ivaniushina V. Alexandrov D. (2022). School structure, bullying by teachers, moral disengagement, and students' aggression: a mediation model. Front. Psychol. 13:883750. doi: 10.3389/fpsyg.2022.883750, PMID: 36148094 PMC9486699

[ref48] Jensen L. A. (Ed.) (2020). The Oxford Handbook of Moral Development: An Interdisciplinary Perspective. Oxford: Oxford University Press.

[ref49] Killen M. Smetana J. G. (Eds.) (2022). Handbook of Moral Development. 3rd Edn. New York: Routledge.

[ref50] Killer B. Bussey K. Hawes D. J. Hunt C. (2019). A meta-analysis of the relationship between moral disengagement and bullying roles in youth. Aggress. Behav. 45, 450–462. doi: 10.1002/ab.21833, PMID: 30900277

[ref51] Kim J. Sijtsema J. J. Thornberg R. Caravita S. C. S. Hong J. S. (2024). Shaping citizenship in the classroom: peer influences on moral disengagement, social goals, and a sense of peer community. J. Youth Adolesc. 53, 732–743. doi: 10.1007/s10964-023-01916-1, PMID: 38091164

[ref52] Kloo M. Thornberg R. Wänström L. (2023). Classroom-level authoritative teaching and its associations with bullying perpetration and victimization. J. Sch. Violence 22, 276–289. doi: 10.1080/15388220.2023.2180746

[ref53] Kollerová L. Soukup P. Gini G. (2018). Classroom collective moral disengagement scale: validation in Czech adolescents. Eur. J. Dev. Psychol. 15, 184–191. doi: 10.1080/17405629.2017.1292907

[ref54] Krause A. Smith J. D. (2023). The interconnected school context: Meta-analyses of the associations between peer aggression involvement and teacher-student relationship closeness. Sch. Psychol. Int. 44, 396–446. doi: 10.1177/01430343221138038

[ref55] Laninga-Wijnen L. Yanagida T. Garandeau C. F. Malamut S. T. Veenstra R. Salmivalli C. (2023). Is there really a healthy context paradox for victims of bullying? A longitudinal test of bidirectional within-and between-person associations between victimization and psychological problems. Dev. Psychopathol., 1–15. doi: 10.1017/S0954579423001384 37990407

[ref56] Lau C. Wong M. Dudovitz R. (2018). School disciplinary style and adolescent health. J. Adolesc. Health 62, 136–142. doi: 10.1016/j.jadohealth.2017.08.011, PMID: 29102555 PMC5803299

[ref57] Luo A. Bussey K. (2023). Moral disengagement in youth: a meta-analytic review. Dev. Rev. 70:101101. doi: 10.1016/j.dr.2023.101101

[ref58] Malti T. Galarneau E. Peplak J. (2021). Moral development in adolescence. J. Res. Adolesc. 31, 1097–1113. doi: 10.1111/jora.12639 34820950

[ref59] Preacher K. J. Curran P. J. Bauer D. J. (2006). Computational tools for probing interactions in multiple linear regression, multilevel modeling, and latent curve analysis. J. Educ. Behav. Stat. 31, 437–448. doi: 10.3102/10769986031004437

[ref60] Sawyer S. M. Afifi R. A. Bearinger L. H. Blakemore S. J. Dick B. Ezeh A. C.. (2012). Adolescence: a foundation for future health. Lancet 379, 1630–1640. doi: 10.1016/S0140-6736(12)60072-5 22538178

[ref61] Sjögren B. Thornberg R. Wänström L. Gini G. (2021a). Associations between students’ bystander behavior and individual and classroom collective moral disengagement. Educ. Psychol. 41, 264–281. doi: 10.1080/01443410.2020.1828832

[ref62] Sjögren B. Thornberg R. Wänström L. Gini G. (2021b). Bystander behaviour in peer victimisation: moral disengagement, defender self-efficacy and student-teacher relationship quality. Res. Pap. Educ. 36, 588–610. doi: 10.1080/02671522.2020.1723679

[ref63] Swedish Education Act, SFS 2010:800. (2010). Available at: https://rkrattsbaser.gov.se/sfst?bet=2010:800 (Accessed April 16, 2024).

[ref64] ten Bokkel I. M. Roorda D. L. Maes M. Verschueren K. Colpin H. (2023). The role of affective teacher–student relationships in bullying and peer victimization: a multilevel meta-analysis. Sch. Psychol. Rev. 52, 110–129. doi: 10.1080/2372966X.2022.2029218

[ref65] Teng Z. Bear G. G. Yang C. Nie Q. Guo C. (2020). Moral disengagement and bullying perpetration: a longitudinal study of the moderating effect of school climate. Sch. Psychol. 35, 99–109. doi: 10.1037/spq0000348, PMID: 31804101

[ref66] Thornberg R. (2023). Longitudinal link between moral disengagement and bullying among children and adolescents: a systematic review. Eur. J. Dev. Psychol. 20, 1099–1129. doi: 10.1080/17405629.2023.2191945

[ref67] Thornberg R. Jungert T. (2014). School bullying and the mechanisms of moral disengagement. Aggress. Behav. 40, 99–108. doi: 10.1002/ab.21509 24496999

[ref68] Thornberg R. Jungert T. Hong J. S. (2023). The indirect association between moral disengagement and bystander behaviors in school bullying through motivation: structural equation modelling and mediation analysis. Soc. Psychol. Educ. 26, 533–556. doi: 10.1007/s11218-022-09754-y

[ref69] Thornberg R. Wänström L. Gini G. Varjas K. Meyers J. Elmelid R.. (2021). Collective moral disengagement and its associations with bullying perpetration and victimization in students. Educ. Psychol. 41, 952–966. doi: 10.1080/01443410.2020.1843005

[ref70] Thornberg R. Wänström L. Hymel S. (2019). Individual and classroom social-cognitive processes in bullying: a short-term longitudinal multilevel study. Front. Psychol. 10:1752. doi: 10.3389/fpsyg.2019.01752, PMID: 31417471 PMC6685359

[ref71] Thornberg R. Wänström L. Jungert T. (2018). Authoritative classroom climate and its relations to bullying victimization and bystander behaviors. Sch. Psychol. Int. 39, 663–680. doi: 10.1177/0143034318809762

[ref72] Troop-Gordon W. MacDonald A. P. Corbitt-Hall D. J. (2019). Children’s peer beliefs, friendlessness, and friendship quality: reciprocal influences and contributions to internalizing symptoms. Dev. Psychol. 55, 2428–2439. doi: 10.1037/dev0000812 31436458

[ref73] Turner J. C. Oakes P. J. (1989). “Self-categorization theory and social influence” in Psychology of Group Influence. ed. Paulus P. B.. 2nd ed (Hillsdale, NJ: Erlbaum), 233–275.

[ref74] Walker J. M. T. (2009). Authoritative classroom management: how control and nurturance work together. Theory Pract. 48, 122–129. doi: 10.1080/00405840902776392

[ref75] Wang C. Li B. Zhang L. Liu Y. Xu P. (2022). Prosocial behavior and teachers’ attitudes towards bullying on peer victimization among middle school students: examining the cross-level moderating effect of classroom climate. Sch. Psychol. Rev., 1–14. doi: 10.1080/2372966X.2021.2009313

[ref76] Wentzel K. R. (2002). Are effective teachers like good parents? Teaching styles and student adjustment in early adolescence. Child Dev. 73, 287–301. doi: 10.1111/1467-8624.00406, PMID: 14717258

[ref9002] White J. Bandura A. Bero L. A. (2009). Moral disengagement in the corporate world. Accountability in Research 16, 41–74. doi: 10.1080/08989620802689847 19247852

[ref78] Yoo H. N. Smetana J. G. (2022). Distinctions between moral and conventional judgments from early to middle childhood: a meta-analysis of social domain theory research. Dev. Psychol. 58, 874–889. doi: 10.1037/dev0001330, PMID: 35311313

